# The Design of a Single-Bit CMOS Image Sensor for Iris Recognition Applications

**DOI:** 10.3390/s18020669

**Published:** 2018-02-24

**Authors:** Keunyeol Park, Minkyu Song, Soo Youn Kim

**Affiliations:** Department of Semiconductor Science, Dongguk University-Seoul, Seoul 04620, Korea; kj170494@dongguk.edu (K.P.); mksong@dongguk.edu (M.S.)

**Keywords:** Analog-to-digital converter, CMOS image sensor, edge detection, iris recognition, XOR

## Abstract

This paper presents a single-bit CMOS image sensor (CIS) that uses a data processing technique with an edge detection block for simple iris segmentation. In order to recognize the iris image, the image sensor conventionally captures high-resolution image data in digital code, extracts the iris data, and then compares it with a reference image through a recognition algorithm. However, in this case, the frame rate decreases by the time required for digital signal conversion of multi-bit digital data through the analog-to-digital converter (ADC) in the CIS. In order to reduce the overall processing time as well as the power consumption, we propose a data processing technique with an exclusive OR (XOR) logic gate to obtain single-bit and edge detection image data instead of multi-bit image data through the ADC. In addition, we propose a logarithmic counter to efficiently measure single-bit image data that can be applied to the iris recognition algorithm. The effective area of the proposed single-bit image sensor (174 × 144 pixel) is 2.84 mm^2^ with a 0.18 μm 1-poly 4-metal CMOS image sensor process. The power consumption of the proposed single-bit CIS is 2.8 mW with a 3.3 V of supply voltage and 520 frame/s of the maximum frame rates. The error rate of the ADC is 0.24 least significant bit (LSB) on an 8-bit ADC basis at a 50 MHz sampling frequency.

## 1. Introduction

**Figure 1:**
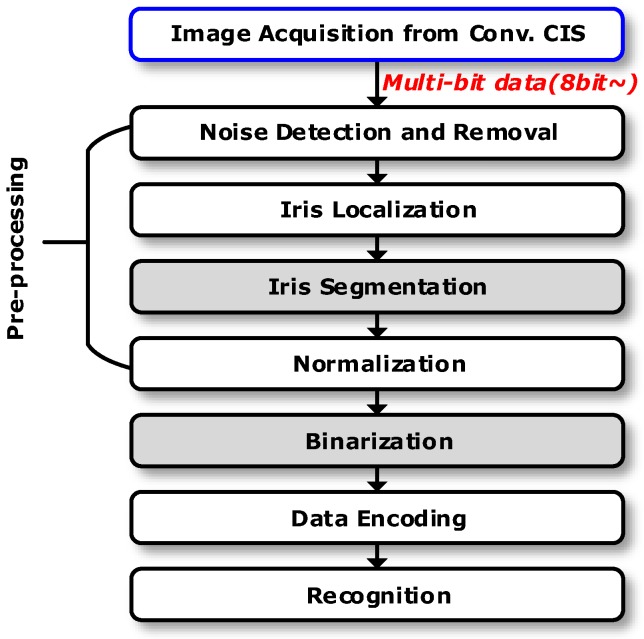
A conventional iris recognition process, adopted from [4].

In recent years, the development of various information technologies has increased the importance of security systems for mobile devices. The security level of biometrics such as voice, iris or retina pattern, and fingerprints is quite high since these cannot be duplicated or stolen [1]. Iris recognition has been developing rapidly for unlocking the passcode of mobile phones [2,3]. Conventionally, in order to use the iris as a biometrics, an iris recognition algorithm must consist of image acquisition, pre-processing, iris image enhancement, binarization, and recognition processes as shown in Figure 1 [4]. A CMOS image sensor (CIS) plays an important role in capturing the iris images to be analyzed and converted. First, a photo detector array (i.e., a pixel array) in the CIS converts an amount of light into corresponding voltage, which is transformed into a multi-bit digital code through an analog-to-digital converter (ADC). The digital code (i.e., the output of the ADC) with the iris image data is compared to the reference image using algorithms in the application processor (AP) [5,6]. However, in order to carry out iris recognition using the conventional method, a high-resolution and high-speed CIS is necessary since certain measurement conditions under which iris patterns are obtained are often not stable; for example, a user’s hand may shake, or the angle at which a photograph is taken may be irregular [7]. Once the iris images are acquired using CIS, the iris region is determined through the boundary of the pupil, iris, and sclera through the multi-bit image data output from the CIS. Since the detected iris region has a circular shape expressed by polar coordinates, it must be rearranged into a rectangle shape that represents the iris pattern as a single bit through a differential operation mask [7,8]. In addition, for the purpose of iris recognition, it is important to recognize a unique pattern. For example, when the comparison image and the measurement environment are different, the pixels in the CIS at the same point may have different values. Therefore, in the conventional iris recognition method, the CIS provides only high-resolution image data, re-analyzes the image data, converts the data into iris pattern data, and compares it with the reference image [9]. However, since the output of the conventional CIS is multi-bit data (higher than 8–10 bit) after the analog-to-digital conversion, high image resolution and slow ADC conversion time are unavoidable, leading to a decreased frame rate [10,11].

Therefore, in order to obtain an accurate iris recognition function, especially iris segmentation function using a basic structure of existing CIS, we propose a single-bit CIS that can display an iris segmentation pattern with an edge detection block to obtain a single-bit output instead of multi-bit output. The contents of the paper are as follows. Section 2 discusses the proposed single-bit CMOS image sensor for iris recognition, as well as its circuit design and implementation. The experimental results and conclusion are summarized in Section 3 and Section 4, respectively.

## 2. Design of the Proposed CIS for Iris Segmentation Using Edge Detection Block 

### 2.1. The Proposed Single-Bit CIS with Iris Recognition Algorithm

**Figure 2:**
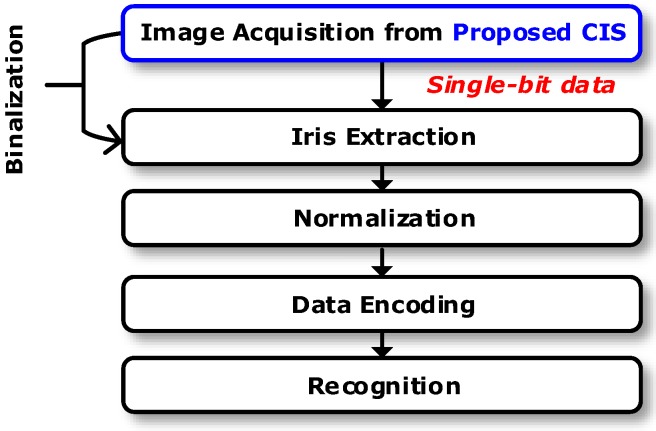
The proposed CMOS image sensor (CIS) with an iris recognition algorithm.

Figure 2 shows the proposed CIS with the iris recognition algorithm. Unlike a conventional iris recognition algorithm using CISs with multi-bit output as shown in Figure 1, the output of the proposed CIS is single-bit after edges are detected. Therefore, the process of converting a high-resolution image data into single-bit using a differential operation mask (i.e., iris segmentation) can be excluded. Such segmentation mask is typically computationally demanding and affected by the measurement environment. Therefore, the proposed single-bit CIS could help reduce the randomness of the decision of biometric algorithms. Furthermore, since the single-bit output of the proposed CIS performs iris segmentation and binarization, the process of iris recognition can be less complicated compared to the conventional iris recognition process in Figure 1. In addition, since the data processing of the CIS is also made up of a single bit without an ADC, it was expected that the operation speed (i.e., frames per second, fps) would be faster than that of the conventional image sensor and that the area and power consumption for the circuits would be smaller. It should be noted that the proposed CIS can employ the same processes of normalization, data encoding, and iris recognition as those of conventional iris algorithms [3,4].

### 2.2. The Operation of the Proposed CIS

**Figure 3:**
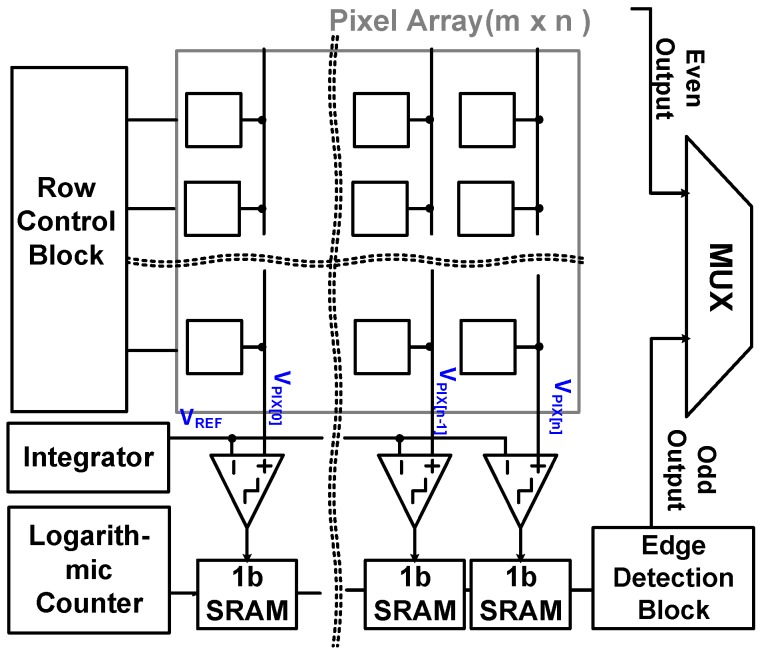
A block diagram of the proposed iris recognition sensor.

**Figure 4:**
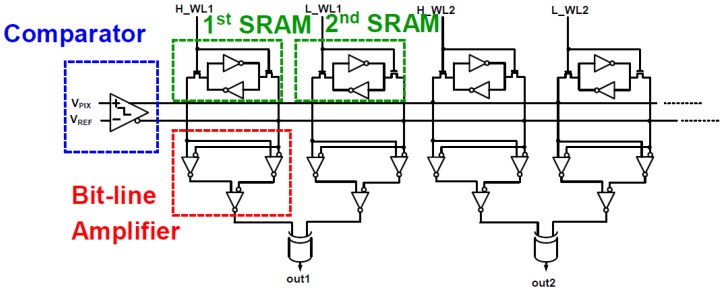
A single column schematic of the proposed CIS.

**Figure 5:**
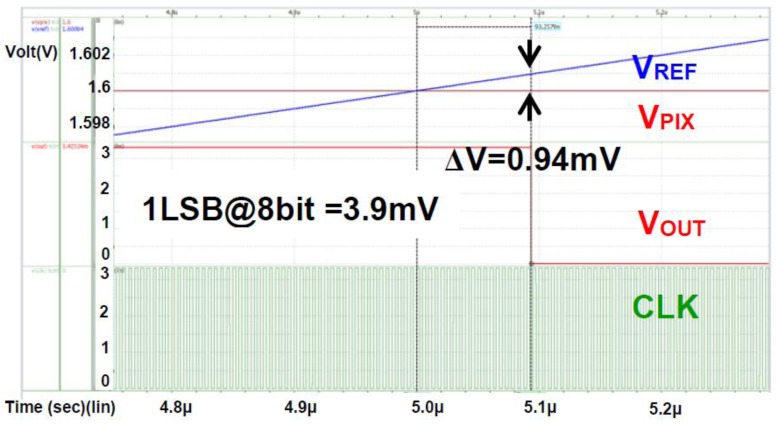
Simulation results of the 1-bit clocked comparator with V_PP_ = 1 V, 50 MHz of sampling frequency.

Figure 3 shows the block diagram of the proposed CIS in this paper. The sensor consists of a pixel array (174 × 144), 1-bit static random access memories (SRAMs), edge detection block using an XOR gate, a row control block, and an output multiplexer (MUX). As in conventional CIS, the pixels in this paper convert the amount of light into corresponding voltages, which are the input for the 1-bit comparator, V_PIX_. The pixel voltage (V_PIX_) acquired from the pixel array and the ramping voltage (V_REF_) acquired from an integrator are applied to the comparator as shown in Figure 4. Figure 5 shows the simulation results of the comparator with 1 V of V_PP_ and 50 MHz of sampling frequency. The comparator can detect 0.94 mV of an offset error, which is smaller than 1 LSB of the 8-bit ADC (~3.906 mV = 1 V/ 2^8^), leading to a 0.24 LSB error rate (i.e., 0.94 mV/3.906 mV). Table 1 shows the Monte Carlo simulation result of the comparator with different process conditions (nn, ff, ss). Under the worst condition, we observed a 0.65 LSB error rate at maximum. Furthermore, owing to the single-bit CIS operation, only 600 clk (i.e., 12 μs at a 50 MHz sampling frequency) is required for operating pixels, comparing, and reading out 1-row, including horizontal blank time. Therefore, the maximum frame rate of the proposed CIS has a QCIF resolution (174 × 144 pixels) is 520 (i.e., 1/(0.2 μs × 600 × 160), including the number of rows and the vertical blank time), leading to an increase in the accuracy of iris recognition. In general, the frame rate of conventional CISs used for iris recognition is about 60 fps (frames per second) with a 2 M pixel resolution and 100 fps with VGA resolution when the conventional CISs are operated with a 10-bit ADC [12,13].

**Figure 6:**
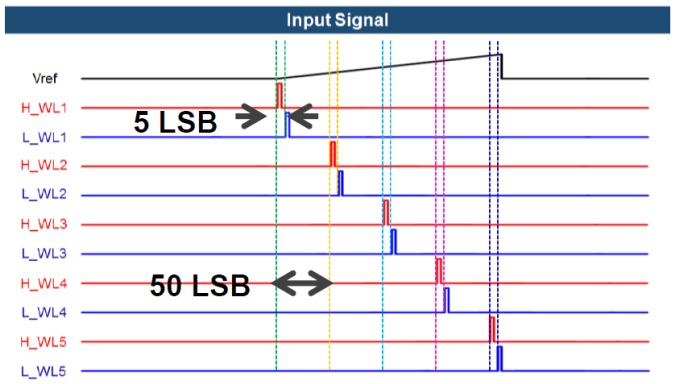
Example of static random access memory (SRAM) word-line signals.

**Figure 7:**
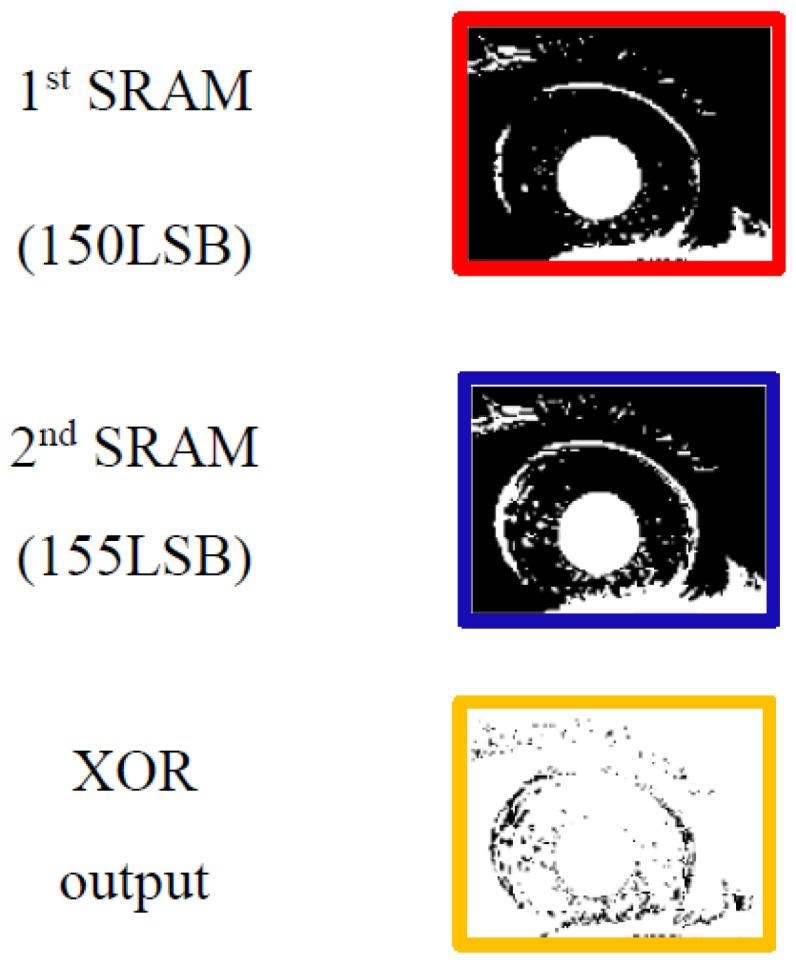
XOR output of two different images from dual-SRAM.

**Figure 8:**
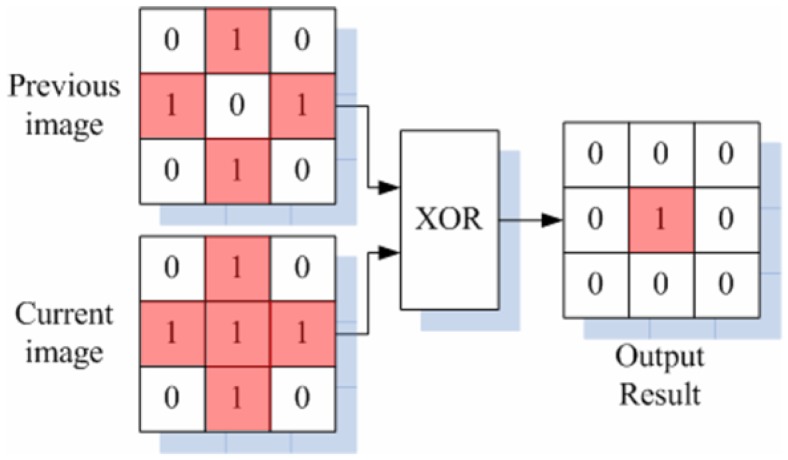
Edge (boundary) detection with the XOR gate.

After the comparison is finished, the output of the comparator is applied to the bit line of the SRAM through the buffer. The SRAM stores data through a separate signal applied to a word line, such as H_WL1 and L_WL1 in Figure 4. Figure 6 shows the signal applied to each word line of the SRAM. A pair of H-word lines and L-word lines in Figure 4 is applied with a pulse having a spacing of 5 LSBs (between H_WL1 and L_WL1) based on 8 bits relative to the rise time of the ramping signal (V_REF_). When the word line is applied at the time at which the pulse is applied, the outputs of the comparators are stored in the cross-coupled inverters in SRAM. The bit lines of the pair of H-word line SRAM and L-word line SRAM are connected to an amplifier (which acts as a sense amplifier in the memory circuits) to amplify the signal, and the output is applied to the input of the edge detection block. It should be noted that we chose 5 LSBs between two word lines to detect a 5 LSB difference. For example, if there is an iris image difference between 150 and 155 LSBs that are stored in the 1st and 2nd SRAMs, respectively, as shown in Figure 7, the boundary can be detected using an XOR gate, which is the edge detection block. Figure 8 shows the process of edge detection using XOR. The information of each pixel is converted to ‘1’ by the XOR gate and detects the boundary (i.e., the edge). The data of each SRAM input through the comparator has different data values according to the timing of the applied word line. A pixel with different data in another word line signal, as in Figure 8, represents the boundary of the 5 LSB difference between the H- and L-word lines. This makes it possible to represent the boundaries of the image in a single bit. Therefore, one piece of boundary information can be obtained through a pair of H and L-SRAMs, and five pieces of boundary information can be obtained through five H- and L-SRAM pairs. At this time, each pair of H- and L-SRAMs represents the boundary information for a 40 LSB difference according to the word line signal.

### 2.3. The Proposed Logarithmic Counter for Word Line Signals

**Figure 9:**
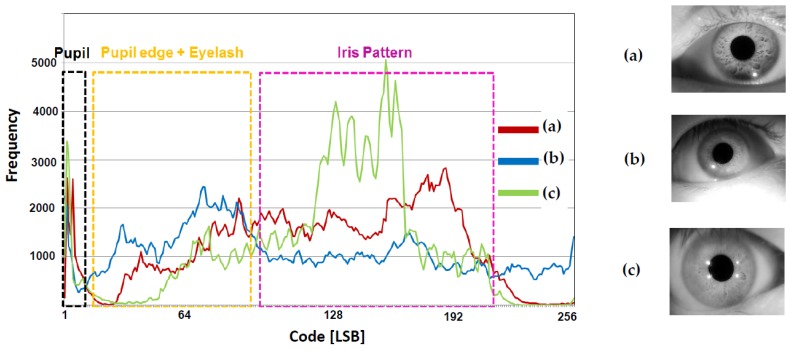
Pixel data distribution at an 8-bit image.

**Figure 10:**
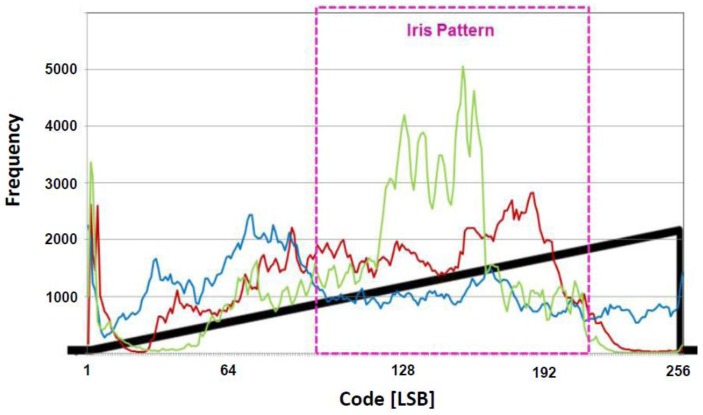
Iris region at the ramp signal (V_REF_).

**Figure 11:**
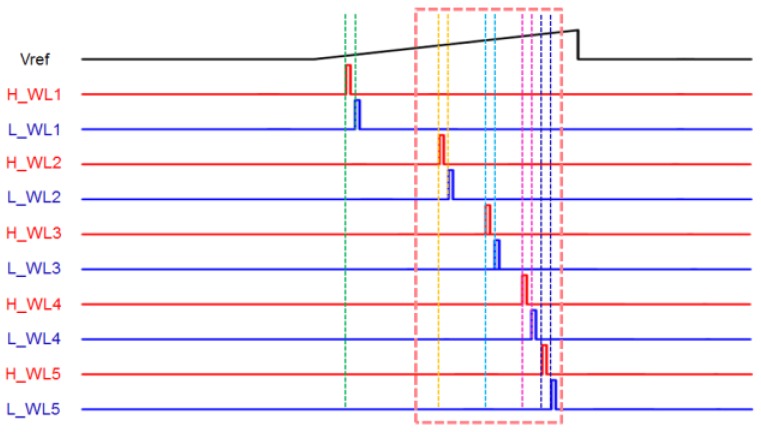
SRAM data region when pulse signal gap decreases exponentially.

The proposed CIS stores data according to when the word line is applied in the SRAM. Therefore, the iris recognition efficiency improves only when the pulse of a word line is applied at a portion where an iris pattern exists at a certain V_PP_. Figure 9 shows the distribution of 8-bit codes, which converts an image taken by a 10-bit CIS camera into a monochrome image. It should be noted that we extract the iris region from the captured original eye images to make the histogram. The lowest code is distributed in dense pupils, and the pupil–iris boundaries and eyelashes are distributed in the middle and lower codes. Most of the iris patterns are distributed in the upper and lower codes, and the uppermost code is mostly reflected light. That is, in the word line signal as shown in Figure 6, since the iris pattern and the other data area are stored, the efficiency might be low. Furthermore, depending on the image measurement environment, the data of the pixel itself may also be distributed as a code that is higher or lower than the distribution in Figure 9, so the word line signal cannot be set as constant. In the proposed CIS, not only the iris pattern but also the boundary of the iris region must be detected. Therefore, it is necessary to store a small amount of data in the case of a pupil with clear boundaries and to store a large amount of data in an area where a large number of iris pattern data exists. Figure 10 shows the region where the iris pattern exists in the ramp signal applied to the comparator. As shown in Figure 7, when the pulse signal is applied, it is difficult to store a large number of regions in which the iris pattern mainly exists. Figure 11 shows a case where the interval of the word line pulse applied to each SRAM pair decreases exponentially. The fewer data in the lowest region in which the pupil is located is stored, and the data in the upper and lower regions in which the iris pattern is located can be stored intensively. In addition, the sensing area can be optimized by changing the exponential function according to the measurement environment.

## 3. Experimental Results

### 3.1. Layout of the Proposed CIS

**Figure 12:**
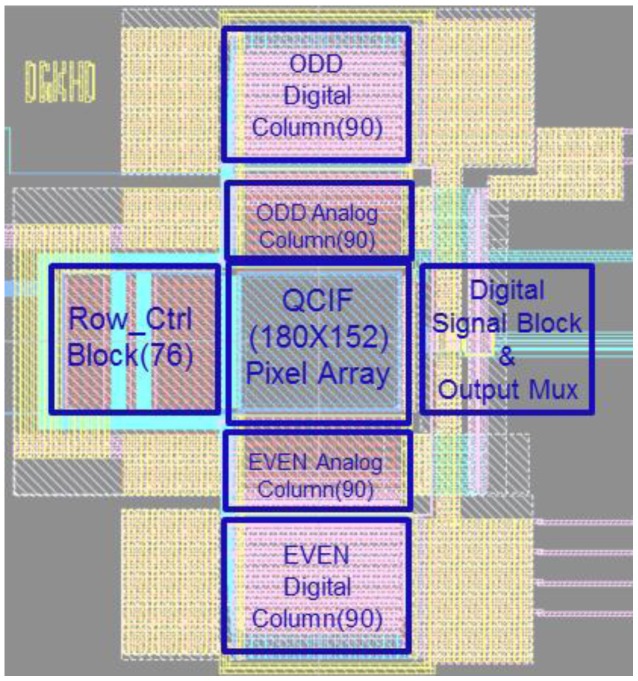
Layout of the proposed CIS.

The proposed CIS has a QCIF resolution (174 × 144 pixels) with a 3.3 V supply voltage and a 0.18 μm 1-poly 4-metal CIS process. Figure 12 shows the layout of the proposed CIS using a 1-bit comparator with XOR gates to detect the edge.

### 3.2. Measurement Results

**Figure 13:**
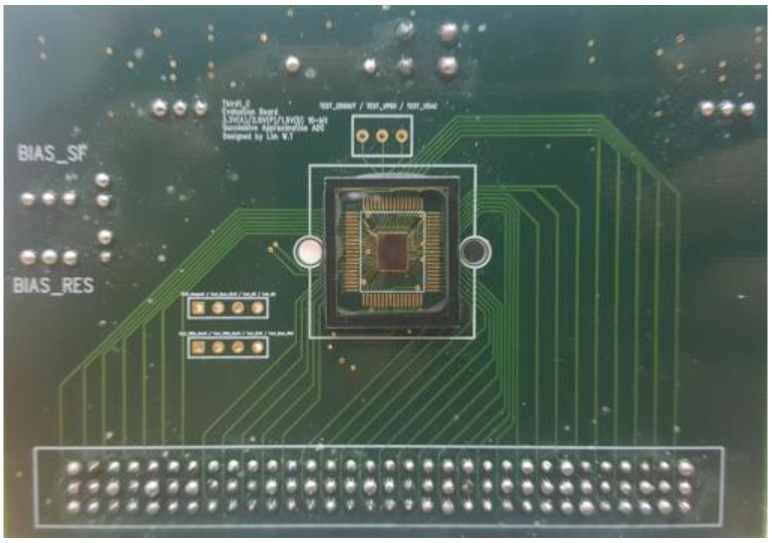
Fabricated chip on a PCB board.

The proposed CIS is fabricated as shown in Figure 13. The total area of the chip, excluding I/O pads, is 1.72 mm × 1.65 mm, and the effective area is 2.84 mm^2^. The total power consumption is 2.8 mW with a 3.3 V supply voltage with 60 fps.

**Figure 14:**
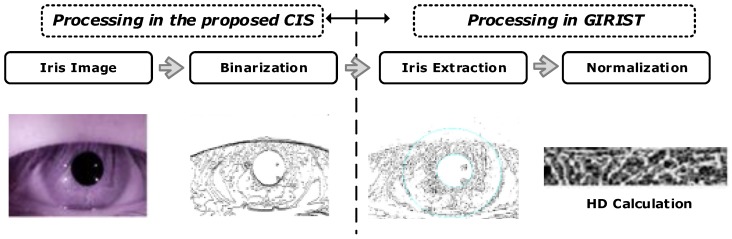
Iris recognition processing using the proposed CIS.

**Figure 15:**
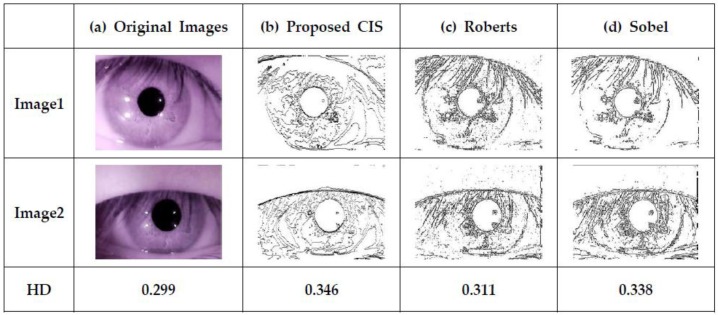
Images based on the conventional iris recognition algorithms and the proposed CIS.

Figure 14 shows the iris recognition processing with the proposed CIS. In order to demonstrate the iris recognition process using the proposed CIS, we adapt software for the iris recognition system [14], GIRIST [15], which can compute container code recognition (CCR) to evaluate the accuracy of identification. First, single-bit image outputs are obtained after edges are detected from the proposed CIS. The result is the same as that of “binarization” in a conventional recognition algorithm, since a raw image is converted to a single-bit output. Then, the result of binarization is the input of the GIRIST that detects the iris region and normalizes the iris data to evaluate hamming distance (HD). The GIRIST judges that the different iris images are from the same person only when the threshold of HD is less than 0.36. Figure 15 shows our test images from the proposed CIS compared to other algorithms used, such as those of Roberts and Sobel [16], using GIRIST. In order to validate GIRIST, we ran GIRIST with two original images (8-bit raw images) of an eye as shown in Figure 15a. GIRIST judged them as “same eye” since the HD was 0.299. Figure 15b shows two single-bit images from the proposed CIS, resulting in 0.346 of HD. Figure 15c,d shows 0.311 and 0.338 of HD using Roberts and Sobel, respectively. From the comparison results, we observe that the HD of the proposed single-bit CIS, compared to Roberts and Sobel, is slightly smaller. However, the proposed CIS also works well for iris recognition since the HD is 0.346 (which is smaller than 0.36), resulting in a “same eye” judgment from GIRIST. Therefore, the single-bit images of the proposed CIS can be used for iris recognition without a heavy algorithm to perform iris segmentation and binarization, finally leading to a reduction in chip area and power consumption.

The full chip was measured using a field-programmable gate array (FPGA) board to generate the control signals required for a clocked comparator, memory block, and other operations. The signal generated by Xilinx was applied to the design circuit through the motherboard using the FPGA board, and the data was then displayed on a computer screen using the universal serial bus (USB) interface. Table 2 provides the detailed specifications of the proposed CIS.

## 4. Conclusions

In this paper, we implemented a single-bit CIS that detects iris patterns in an image sensor and converts them into a single-bit code that is applicable to an algorithm for iris recognition. Using the proposed CIS, we successfully obtained images that are sufficient to judge an iris even with the naked eye by simple circuit structure using a 1-bit comparator and XOR gates rather than a complicated high-resolution ADC and extra circuits for an iris segmentation algorithm. Furthermore, we achieved the lower power consumption and improved frame rates compared to the conventional CIS since the proposed CIS does not require the timing for ADC operation, which cannot be eliminated in conventional CIS structures. We believe that the proposed circuit structure of CIS for iris recognition applications can eventually lead to ultra-low power sensors that can be useful for various biometrics applications.

## Figures and Tables

**Table 1:** Monte Carlo simulation results of the comparator.

Process	Open Loop Gain [dB]				Unity Gain Frequency [MHz]			
	Max.	Min.	Mean	STD.	Max.	Min.	Mean	STD.
ff	34.82	25.4	29.74	1.38	32.3	28.12	30.36	0.81
nn	34.02	25.1	29.61	1.34	23.91	22.07	23.08	0.35
ss	32.79	24.9	29.55	1.30	19.27	17.7	18.60	0.38

Max.: Maximum, Min.: Minimum, STD: standard deviation.

**Table 2:** Performance summary of the proposed CIS.

Process	0.18 μm 1P4M CMOS process
Chip size	2.35 mm × 2.35 mm(5.53 mm^2^)
Core size	1.72 mm × 1.65 mm(2.84 mm^2^)
Resolution	QCIF (174 × 144)
Pixel type	4-shared 4T-APS
Supply voltages	3.3 V(Analog)/1.8 (Digital)
ADC resolution	1 bit (8-bit ADC-comparable accuracy)
Power consumption	2.80 mW @ 60 fps 12.36 mW @ 520 fps
Maximum frame rate	520 fps
